# Materials Development for Next Generation Optical Fiber

**DOI:** 10.3390/ma7064411

**Published:** 2014-06-11

**Authors:** John Ballato, Peter Dragic

**Affiliations:** 1 The Center for Optical Materials Science and Engineering Technologies (COMSET) and the Department of Materials Science and Engineering, Clemson University, Clemson, SC 29634, USA; 2 Department of Electrical and Computer Engineering, University of Illinois at Urbana-Champaign, Urbana, IL 61801, USA; E-Mail: p-dragic@illinois.edu

**Keywords:** optical fiber, high energy lasers, stimulated Brillouin scattering, stimulated Raman scattering

## Abstract

Optical fibers, the enablers of the Internet, are being used in an ever more diverse array of applications. Many of the rapidly growing deployments of fibers are in high-power and, particularly, high power-per-unit-bandwidth systems where well-known optical nonlinearities have historically not been especially consequential in limiting overall performance. Today, however, nominally weak effects, most notably stimulated Brillouin scattering (SBS) and stimulated Raman scattering (SRS) are among the principal phenomena restricting continued scaling to higher optical power levels. In order to address these limitations, the optical fiber community has focused dominantly on geometry-related solutions such as large mode area (LMA) designs. Since such scattering, and all other linear and nonlinear optical phenomena including higher order mode instability (HOMI), are fundamentally materials-based in origin, this paper unapologetically advocates material solutions to present and future performance limitations. As such, this paper represents a ‘call to arms’ for material scientists and engineers to engage in this opportunity to drive the future development of optical fibers that address many of the grand engineering challenges of our day.

## 1. Introduction

Light today enables an annual global economic impact of about $7.5 trillion (USD) [1], which is approximately half of the gross domestic product (GPD) of the United States; and light’s economic value is growing. The three largest market sectors relying on light-based products are those associated with telecommunications, transportation, and biotechnologies [2]. The telecommunications sector constitutes over 50% of the economic value. Core technologies enabled by light include e-commerce, Internet music and movies, and information routing and storage. The transportation sector includes laser-based manufacturing (e.g., precision cutting, drilling, and welding) of automobiles and airplanes. The biotechnological sector includes laser and optics-based diagnostic and surgical systems as well as Laser-Assisted in-situ Keratomileusis (LASIK) eye surgery, blood testing, and gene sequencing [2]. Smaller by market value but critical nonetheless are laser-based defense, security, and sensor systems, which will continue to drive technology development especially at “specialty” (high value and performance, low volume) levels. 

Another way to view this cumulative effect on global goods and services is that light-based solutions exist (or could reasonably exist with further advancements) for many of the technology platforms identified as being most central to society over the next decade [3]. Despite this centrality of light to the many modern conveniences there has been decades-long erosion in the appreciation given to the materials as enablers of such technologies [4]. Modern and prototypic future systems that are prescribed as solutions to present performance limitations are strongly driven by ‘structural’ (geometric) as opposed to material considerations. Perhaps the best exemplars of these are photonic crystal fibers (PCFs) which are transversely periodic arrays of air holes surrounded by glass that run longitudinally down an optical fiber. Such designs range from the fairly straight-forward (note that the word “simple” was not used) to the intricately absurd; at least with an eye towards scalability and industrial, albeit low volume, manufacturing. Yet while PCFs can force light to do its bidding in a wide variety of useful ways, many phenomena, such as reduced nonlinearities, can be accomplished more simply through materials; or so this paper plans to promote and prove.

In reality and with practicality in mind, the true solution is both. It takes little more than opening a basic physics book to remember that properties are dependent upon both shape and substance; e.g., the capacitance of a capacitor—amongst the most elementary electronic devices—is dependent upon its geometry (area and thickness) and the material (specifically its permittivity) from which it is made. However, we, the materials community, are at a cross-road. A recent (albeit US-centric) study by Corning Incorporated [5] concludes by noting that “… less than one-quarter of students at US universities who are doing research in glass science are studying systems that would make them well prepared for a future career in the glass industry. In our experience … students with expertise in glass families that are industrially relevant … are more likely to be hired into a position in industry and also require less on the job technical training after being hired.” The authors of that work go on to identify a very rich range of problems that materials scientists and engineers can work on (and get employed to solve!) that would have significant societal benefits.

Accordingly, this paper seeks to amplify further the recommendations of two recent works [4,5] by providing a review of materials-related solutions to power-scaling in high energy optical fiber laser-based systems. Given that the lowest threshold nonlinearities that presently limit performance are stimulated Brillouin scattering (SBS) and stimulated Raman scattering (SRS), these phenomena will be used as primary exemplars. Also to be discussed are higher order mode instabilities (HOMI) and management of the nonlinear refractive index. It is the authors’ hope that this work, along with [4,5], will help to reinvigorate the global materials community to rejoin the development of next generation optical fibers. 

## 2. Results and Discussion

The intensity of light in both high-power continuous wave (CW) and high power-per-unit-bandwidth pulsed laser systems is sufficient to induce nonlinearities in the material response to the propagating electromagnetic field. The result is a number of nonlinear and parametric photonic processes that are widely used or alternatively considered to be parasitic to system performance [6], including Raman scattering (or stimulated Raman scattering, SRS), Brillouin scattering (or stimulated Brillouin scattering, SBS), Self-Phase Modulation (SPM), and Four-Wave Mixing (FWM). Each of these is described in greater detail in Section 2.1 as are their materials’ origins.

Since these parasitic effects are intensity-dependent, one approach to their control is to spread the optical power out over a larger cross-sectional area thereby reducing the effective power-per-unit area. In practice, this has led to so-called “large mode area” (LMA) fibers where the dimension of the core in which the light is propagating is expanded relative to more conventional designs. However, such large core sizes, coupled with typical fiber numerical apertures (NAs) which together generally render such fibers ‘few-moded’ almost always promote multimode guidance such that the beam quality of the resultant laser light is deteriorated. Several methods to overcome this obstacle have been proposed and implemented. A few of these are outlined next. 

In principle, restricting the light launch conditions such that only the fundamental optical mode (FOM) is excited is one way to achieve the highest beam quality [7] available from such fibers. However, mode coupling is brought about through fiber bends, packaging, and environment, leading to degradation in the quality (or M^2^) of the beam in most practical situations. Therefore, an early approach to overcoming the inevitable multimode nature of the beam in these LMA fibers was the discovery that via careful bending of the fiber, higher order modes (HOMs) could be ‘peeled away’ from the core [8] leaving what effectively amounts to single mode operation. The technique calls for coiling the fiber into a preselected diameter such that the differential loss between the fundamental mode and HOMs is large. In this way, the fundamental mode dominates the laser characteristics and captures essentially all of the available gain. It too is possible to design a microstructured fiber for ‘endlessly single mode operation’ [9] with extremely low effective NA, but the intolerance of such fibers to bending has largely confined them to the rod-like regime, with the resultant lasers more closely resembling a cross-over of sorts between bulk and fiber lasers. In the restricted mode launch regime, it was subsequently found that excitation of a HOM in a few-moded fiber offered increased robustness of the mode propagation which was coupled with the added benefit that the HOMs have larger mode areas than the FOM [10].

Other “effectively” single mode LMA fibers were also subsequently developed to force what otherwise is a multimode design into behaving like a single mode fiber. As with the simple fiber coiling method described above, the goal is to provide some sort of differential loss mechanism such that the HOMs experience vastly greater propagation losses than the FOM. Hence the FOM will experience the highest gain and dominate the modal distribution at the laser output. A few recent fiber designs include the chirally-coupled core fiber (HOMs are coupled to and shed from one or more satellite waveguides that form a helix around the fiber core) [11], photonic bandgap Bragg fiber (where only the FOM propagates with low loss) [12], solid photonic bandgap fibers (where bending loss is exacerbated on HOMs relative to the FOM) [13], leakage channel fibers (wherein all modes are lossy, but the loss is minimized for the FOM) [14], and selectively-doped triple clad fibers (where the FOM has the highest overlap with the active region in the fiber) [15]. While there is clearly no shortage of methods utilized to improve the beam quality from an LMA fiber, such fibers generally are quite complex in their cross-sectional geometries. Furthermore, while effectively single mode operation does result, there is a power threshold above which single mode operation is unstable [16,17]. This ‘modal instability’ (MI) or ‘higher order mode instability’ (HOMI) yields a time varying output beam profile that negates many of the benefits of large mode areas fibers. 

The authors have recently articulated a different approach to mitigating and, in some cases, completely removing from consideration, these parasitic effects based on the judicious choice of materials rather than on fiber geometry [4]. Specific examples are provided in Section 2.2 below. However, it is necessary first to describe the interaction of light with the optical material (fiber) through which they propagate and how such nonlinear and/or parasitic phenomena arise. Only then can materials-related solutions be more fully envisioned and appreciated.

### 2.1. The Materials Physics of Linear and Nonlinear Optical Phenomena

Depending on the application, nonlinear phenomena in optical fiber can be useful or deleterious. From the perspective of achieving high powers from active optical fiber systems, these processes are generally parasitic in nature. They give rise to significant limitations to power scaling and tremendous effort has been made in suppressing them (or in other words, elevating the threshold power at which these processes become significant). Since these processes are intensity-dependent, a larger mode diameter in an optical fiber usually implies a higher ‘turn-on’ threshold for these interactions. As described above, this has led to a number of fiber designs aimed at increasing the mode field diameter (MFD) in an optical fiber while maintaining robust single mode operation. While nonlinear effects can be controlled to some extent with proper waveguide design, each of these processes can be described mathematically via a combination of materials properties that cooperate to define a ‘gain’ or ‘gain coefficient’. With careful selection of precursor materials, it is possible to tailor or design the composition such that these gain coefficients are reduced to acceptable levels for a given application.

The first nonlinear process investigated is stimulated Brillouin scattering (SBS). SBS is an interaction between hypersonic (thermally excited) acoustic waves and the optical signal in a fiber. Brillouin scattering begins as a spontaneous process with optical back-scattering most efficiently taking place from acoustic waves that are Bragg-matched to the optical wave. Simply put, the acoustic wave is a periodic longitudinal pressure, and therefore also density, variation. The spatially modulated density of the material then corresponds to a spatially modulated refractive index. Via electrostriction, the interference between the forward-propagating signal and back-scattered light feeds the acoustic (pressure) wave. This ‘positive feedback’ process increases in efficiency as the power is increased until ‘threshold’ is reached wherein the acoustic wave becomes a highly-efficient reflector to the optical signal. At this point Brillouin scattering is in the stimulated state. In general, SBS limits the amount of light per unit bandwidth that can be transmitted down or generated in an optical fiber. As such, it typically has the lowest threshold of all the nonlinear processes in narrow linewidth systems.

The Brillouin gain coefficient for a given optical wavelength (λ) is given to be *g*_B_ = 2π*n*^7^*p*^2^_12_/cλ^2^ρ*V_a_*Δ*ν*_B_ ([18], which has units of m/W), where *n* is the refractive index, *p*_12_ is a Pockels photoelastic constant, *c* is the speed of light, ρ is the mass density, *V*_a_ is the acoustic velocity, and Δ*ν*_B_ is the Brillouin spectral width (which is inversely proportional to the acoustic damping time). Given the dependencies of the Brillouin gain on these physical parameters, beneficial materials properties for the suppression of SBS include: (1) a large acoustic velocity; (2) relatively low index; (3) relatively large mass density; (4) large Brillouin spectral width (*i.e*., large acoustic attenuation coefficient); and (5) low *p*_12_. Unfortunately, a large density typically suggests a relatively large refractive index, so the density and index tend to mitigate one another with respect to *g*_B_. Regardless, a clear guideline with selection criteria can be identified for choosing materials for reduced Brillouin gain.

To model the system, an additive approach based on the work of Schott and Winkelmann [19,20] is utilized to calculate each of the terms in this equation for a mixed multicomponent glass (such as a germanosilicate glass). Specifically, these quantities are 

(a) the acoustic velocity 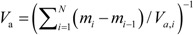; 


(b) the mass density 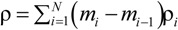; 

(c) the refractive index 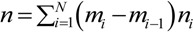; and 

(d) the Brillouin spectral width (Δ*ν*_B_),


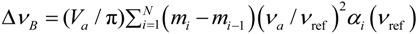

which is proportional to the acoustic attenuation coefficient α (units of m^−1^). The scaling term (*ν_a_*/*ν*_ref_)^2^ accounts for the usual frequency-squared dependence of the intrinsic linewidth, while α is always specified at a reference acoustic frequency *ν*_ref_. It is important to note that it is possible (but seemingly rare) for the scaling term to be a sub-square function [21], depending on the material and thermal history of the glass. For each calculation (*m_i_* − *m_i_*_−1_) is the volume of constituent *i* in the fiber. The Pockels’ elasto-optic coefficient *p*_12_ is calculated utilizing a similar additive approach, but it is lumped in with the refractive index rather than calculated directly. For details, the reader is asked to refer to Reference [22]. The key point with regard to *p*_12_ is that for a material this value can be either positive or negative. Therefore a combination of materials with photoelastic constants of the former and the latter types can give rise to a composition wherein *p*_12_ is very close to zero. As a result, the possibility of a material with zero Brillouin gain exists. Such negation of certain glass coefficients in order to suppress or eliminate a nonlinear effect can also be applied to HOMI in high power optical fibers. It is well-known that restricted mode launch and careful modal control can give rise to effective single-mode operation in a few-moded fiber utilized in a fiber laser. However, at some sharp threshold power a dynamic randomization of the mode distribution at the laser output is observed (referred to as higher order mode instability, or HOMI), and is believed to be the result of a thermally-induced refractive index grating. The process is believed to be driven in part by d*n*/d*T* [16,17]; the grating forms via a longitudinally-periodic thermal variation in the fiber. It is interesting to point out that HOMI is not a process that can be reduced with a wider MFD, as it becomes more significant of a problem as the core diameter is increased [16,17].

While rendering a gain coefficient zero may be possible for some interactions, this may not be the case for others. An example is stimulated Raman scattering (SRS). Raman scattering is an interaction between an optical wave and optical phonons [18]. In the case of silica-dominated materials systems, the Raman spectrum has a peak gain positioned roughly 13.2 THz from the signal frequency. It can be considered to be a parasitic effect in high-peak-power fiber laser systems where wavelength control is mandatory [23]. The excitation of SRS from spontaneous scattering can lead to wavelength shifts and power instabilities that degrade laser performance, thus presenting the need for its suppression. However, the dependence of the Raman gain is on material properties (Raman cross section, refractive index) that do not appear to necessarily facilitate a zeroing of this value [23]. Instead, materials can be selected such that one or more of the following conditions are met [24]: (1) the material is highly disordered in order to broaden the Raman gain spectrum thereby reducing the peak value; (2) high concentrations of materials with low Raman gain are utilized; and (3) materials are utilized that have Raman spectra with minimal overlap. For (3), a mixture of two materials with similar-strength but not overlapping Raman gain spectra could cut the peak Raman gain coefficient in half.

Processes such as Self-Phase Modulation (SPM) and Four-Wave Mixing (FWM) are results of the dependence of the refractive index on the optical intensity [18]. SPM can be observed in pulsed (time-varying) systems, and it results from an intensity-induced time-varying refractive index (due to the time-varying nature of the signal itself). In multi-wavelength systems, FWM is the transfer of an amount of energy to a new wavelength by the amount that is (plus or minus) the difference in photon energy between two extant optical frequencies. In the case of non-degenerate FWM, if the existing optical frequencies are *ν*_1_ and *ν*_2_ (and *ν*_1_ ≠ *ν*_2_) then the new frequencies will be at 2*ν*_1_ − *ν*_2_ and 2*ν*_2_ − *ν*_1_. Similarly, degenerate FWM can also occur (where *ν*_1_ = *ν*_2_) with new frequency components appearing both to the blue and red side of *ν*_1_. The general effect of these processes is to broaden and modify the optical spectrum, and depending on the application this may be undesirable in high peak power and multi-wavelength optical amplifier systems. Since the dependence of the refractive index is given by *n*(*I*) = *n*_0_+*n*_2_*I* where *n*_0_ is the linear refractive index, *I* is the intensity of the optical signal, and *n*_2_ is the nonlinear refractive index, suppression of these parametric phenomena will require a minimization of *n*_2_.

From the above descriptions, a materials solution to these performance-limiting parasitic phenomena rely on developing optical fibers using materials with reduced (if possible, zero) photoelasticity (*p*_12_), peak Raman gain, nonlinear refractive index, and thermo-optic coefficients. Though one composition that exhibits all of these effects would be ideal, it generally is not necessary since each phenomena has a different threshold condition and so often is application specific. The authors therefore suggest a more thoughtful consideration of the fiber’s compositions based on the specific application and anticipated optical power levels.

### 2.2. Materials Solutions

As noted above, stimulated Brillouin scattering (SBS) and stimulated Raman scattering (SRS) are two of the principal nonlinearities that plague continued power scaling-up in fiber-based laser systems. The sections below provide an overview of selected optical fiber material solutions to mitigate and, in the case of SBS potentially negate entirely, these parasitic effects.

#### 2.2.1. Intrinsically Low Brillouin Gain Glasses and Optical Fibers

The fundamental question at play is less so “what materials can be used to lower the Brillouin gain” but, rather, “do the materials that reduce Brillouin gain (when added into silica) form a sufficiently stable glass to draw into optical fiber?” While this question may seem straight-forward to answer, it is not always the case. Firstly, glass stability is a kinetic consideration and so the method of fabrication needs to be determined. For the purposes of this work, the conventional method of manufacturing will be one of the chemical vapor deposition methods employed in the commercial production of optical fiber; *i.e*., outside vapor deposition (OVD), modified chemical vapor deposition (MCVD), vapor axial deposition (VAD), or plasma-assisted chemical vapor deposition (PCVD). In each case, the combination of temperature cycling between soot deposition and translation of the torch along the forming preform followed by subsequent densification and consolidation (and eventual fiber drawing) leads to a fairly extreme set of time and temperature conditions. Second, for practical reasons, the glass from which the fibers are made must be intrinsically low loss, mechanically robust, and compatible with the other fiber components used to make the fiber-based laser system. These conditions conspire to yield silica and high silica-content glasses as the standard family from which practical optical fibers are made. 

This, of course, is not an impediment as silica is, and has been for millennia, a marvel when it comes to glass stability and properties. However, in returning to the more fundamental question of which glass systems could be used as the core phase in high performance optical fibers, the answer turns out to be a fairly small range of compositions. From a materials perspective, the principal additions to silica in commercial optical fibers are GeO_2,_ which raises the refractive index and can enhance photosensitivity, Al_2_O_3_, raises the refractive index and can enhance the solubility of active rare-earth dopants, P_2_O_5_, which raises the refractive index and reduces glass viscosity, B_2_O_3_, which reduces the refractive index and increases the expansion coefficient, and F, which reduces the refractive index and glass viscosity. Importantly, there are limits: for GeO_2_ [25] and P_2_O_5_ [26], there is a complete solid solution with the base SiO_2_ such that liquid-liquid or solid-solid immiscibility is not problematic and the optimum dopant concentration is mainly determined by its impact on refractive index, thermal expansion, and viscosity. For a surprisingly wide range of other additives into silica, such immiscibilities exist and greatly restrict the compositions from which optical fibers can be made. This material limitation has the added detriment of restricting the range of useful properties that the glass can exhibit. Accordingly, ‘mixing the unmixable’ can only be accomplished through novel manufacturing methods that obviate the combinations of time and temperature that conspire to facilitate the phase instabilities [27]. In the sections that follow, the fibers discussed are made using the “molten core” approach, which is described briefly in Section 3 below and references therein. For the purposes of this Review, suffice it to say that the molten core approach is a super-liquidus (or super-consulate point) process where the fiber is drawn at a temperature that exceeds the melting point (or immiscibility limit) of the core phase so that it is a homogeneous liquid. The cladding glass is selected such that it possesses the appropriate viscosity to draw into high quality fiber at this same temperature. Since the core is molten, and generally reasonably fluid, there is expected to be some dissolution of the cladding glass by the core melt during the fiber drawing process. As such, the core of the resultant fiber, which quenches into a glass as the fiber cools, is an ‘interaction product’ of the initial precursor composition and that of the cladding glass. Given that pure SiO_2_ arguably is the most refractory of the conventional glasses, not to mention naturally compatible with commercial optical fiber components, it tends to be the cladding material of choice. Accordingly, the dissolution of some silica into the core melt during molten core processing has the (generally) added benefit of enhancing glass-forming stability of the resultant core.

In specific regard to suppressing stimulated Brillouin scattering (SBS), the natural consequence of the discussion above is whether or not a material solution exists rather than address the issue by spatially distributing the propagating mode over a larger cross-section of the core, *i.e*., a large mode area approach. As noted above, the Brillouin gain at a given wavelength is dependent upon the refractive index, *n*, Pockels photoelastic constant, *p*_12_, mass density, ρ, acoustic velocity, *V*_a_, and Brillouin spectral width, Δ*ν*_B_, of the material through which the light it transmitted. In terms of glass modifiers to silica, one desires compounds that increase the refractive index (for optical waveguide formation), density, acoustic velocity, and Brillouin line-width while reducing the photoelastic coefficient. Divalent metal oxides, such as the alkaline earths (MgO, CaO, SrO, and BaO) and a range of trivalent metal oxides, such as Al_2_O_3_, Y_2_O_3_, and the lanthanide oxides (Ln_2_O_3_ where Ln are the rare-earth elements) are known to impart the aforementioned features into silica glasses. However, in each case, there are quite limited compositional ranges over which miscibility exists in the silicate melt. A classic example of this, relevant to optical fiber systems, is the Al_2_O_3_-SiO_2_ system, which exhibits a well-known two-phase, liquid-liquid immiscibility region for binary aluminosilicates [28]. Accordingly, the concentration of Al_2_O_3_ that can be homogeneously mixed into SiO_2_ given the processing conditions of conventional optical fiber preforms is about 12 weight percent. Unfortunately, the binary systems Y_2_O_3_-SiO_2_ [29], MgO-SiO_2_ [30], CaO-SiO_2_, SrO-SiO_2_ and BaO-SiO_2_ [31], and ternary systems Y_2_O_3_-Al_2_O_3_-SiO_2_ [32] and MgO-Al_2_O_3_-SiO_2_ [30] also suffer from similar limitations though the exact immiscibility range differs for each system. 

As introduced briefly above, the molten core method offers a powerful route to the direct fabrication of optical fibers using materials that otherwise would be very difficult, if not impossible to form into conventional glass preforms and draw. Given the inevitable dissolution of some cladding glass into the core melt, it is often beneficial to begin with a core precursor composition that possesses more than the amount of a given compound desired in the final fiber. Further, and for completeness, it is worth noting that said core precursor phase can be amorphous or crystalline (single or polycrystalline) since it melts during the fiber draw process and therefore has no “memory” of its starting structure. Accordingly, for fiber possessing high concentrations of the aforementioned alkaline earth oxides and sesquioxides the pure end member was employed. As an example, for high Al_2_O_3_-content fibers, pure sapphire was employed [33]. Similarly for high Y_2_O_3_-Al_2_O_3_, BaO- and MgO-Al_2_O_3_-content fibers, YAG (Y_3_Al_5_O_12_), BaO and spinel (MgAl_2_O_4_) were employed [34,35,36]. Since such compounds, at the concentrations realized, typically phase-separate when mixed with silica, these fibers exhibited a range of useful properties that had not previously been observed including a record low Brillouin scattering [33], an athermal Brillouin frequency condition [33], and the identification of an atensic (zero stress-dependent) Brillouin frequency composition [35].

In specific regard to Brillouin scattering, one particularly interesting opportunity that presents itself by using these novel glass compositions enabled by the molten core method is the ability to realize optical fibers possessing zero, or near-zero photoelasticity. As noted above, the Brillouin gain is dependent on several factors, including a square-dependence on the *p*_12_ photoelastic coefficient. While silica glass possesses a *p*_12_ value that is positive, many of the materials noted above possess also about negative *p*_12_ values. The result is a glass that can balance the positive and negative photoelastic contributions of its constituent compounds to yield a fiber with very low Brillouin scattering. As a case in point, sapphire (Al_2_O_3_) possesses a slightly negative *p*_12_ value (−0.03). When the sapphire melts during the molten core process and some of the silica (*p*_12_ = 0.271) from the cladding dissolves into it, the resulting aluminosilicate core exhibited an SBS gain that was reduced by nearly 20 dB in comparison to conventional glasses [33]. So ‘mixing the unmixable’ via the molten core approach can be very useful for obtaining glasses not previously fabricated yet ones with quite extraordinary properties; particularly as they relate to Brillouin scattering. Table 1 provides the derived values for the BGC parameters from a series of novel material fibers made recently. As can also be seen from Table 1, there is a wide range of tailorability in the parameters influencing the Brillouin gain that judicious choice of materials can affect. 

**Table 1:** Brillouin-Gain-Related Material Parameters from Selected Crystal-Derived All-Glass Optical Fibers.

Parameter	Unit	Silica (SiO_2_)	YAG-Derived Fiber	Al_2_O_3_-Derived Fiber	MgO-Derived Fiber	BaO-Derived Fiber	Yb_2_O_3_-Derived Fiber	La_2_O_3_-Derived Fiber
*V*_a_	m/s	5970	7649	9790	8731	3131	4110	3979
ρ	kg/m^3^	2200	3848	3350	3322	4688	8102	5676
Δ*ν*_B_	MHz	17	253	274	^†^	178	1375	181
*n*	–	1.444	1.868	1.653	1.810	1.792	1.881	1.877
*p*_12_	–	0.271	0.022	−0.03	^†^	−0.33	−0.123	−0.027
Reference	–	22	34	22,33	36	35	37	49

† Not able to be measured from fabricated fibers.

#### 2.2.2. Intrinsically Low Raman Gain Glasses and Optical Fibers

**Figure 1:**
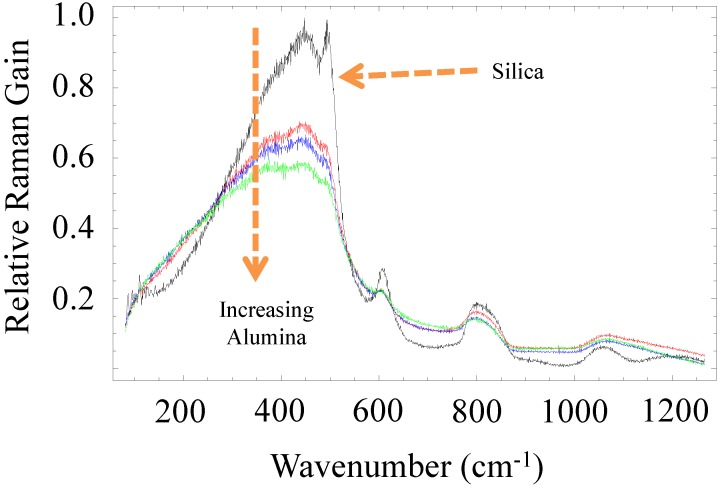
Raman gain spectra (normalized to silica) measured from the sapphire derived fiber for three different alumina concentrations. The spectrum appears to broaden and get weaker relative to the cladding (silica, black line).

Unlike Brillouin gain, Raman gain is not linked to material properties that could be potentially driven to zero by choice of composition. However, by analogy to LMA fibers where the propagating optical mode is spread out over the fiber cross-section to reduce its intensity at a given spatial location, the distribution of the glasses’ bond energies can be broadened thereby reducing the effective Raman gain at a given wavelength. As a case in point, all-glass optical fibers derived from yttrium aluminum garnet (YAG) were fabricated [24]. It was found that the Raman peak at 440 cm^−1^, which is associated with the Si–O–Si stretching mode, did not change position with core glass composition but was much broader spectrally likely due to a larger distribution of Si–O–Si bond angles in these more highly modified glasses. The defect line peaks at 490 cm^−1^ (D_1_) and 600 cm^−1^ (D_2_), which are attributable to the 4- and 3-member ring ‘breathing’ modes respectively, diminished with the combined (Y_2_O_3_ + Al_2_O_3_) content in the resultant glass though their spectral position did not change suggesting that yttria and alumina may lessen the number of ring structures in the glass. These same trends were also later observed for the sapphire-derived (high alumina content) fiber. Raman spectra for the YAG-derived fibers can be found in [24] while Figure 1 shows the set of Raman spectra obtained for the sapphire-derived fiber described above. As mentioned, the features of the spectra trend in ways similar to the YAG-derived fiber. In fact, the Raman gain spectra for the YAG- and sapphire-derived fibers are nearly identical, except that the sapphire-derived fiber appears to be missing the line observed at 950 cm^−1^ (and attributed to the oxygen hole center [24]) from the YAG-derived fibers [37].

The spectra obtained suggest that the core material possesses much more disorder than the pure silica cladding glass. As a consequence of these modifications to the glass structure, the spontaneous Raman scattering intensity from the yttrium-aluminosilicate fibers was found to increase linearly with increasing silica content [24] with a reduction of about 3 dB measured for a silica content of about 67.5 mol%. This trend is similar for the sapphire-derived fiber, with the concentrations of alumina being 26.9, 30.8, and 41.2 mol% for the three aluminosilicate fibers and a maximum reduction in Raman gain of about 2.5 dB for the highest alumina content (see Figure 1). While the reduction in Raman gain can partly be attributable to the replacement of silica with materials of relatively lower Raman gain, it is interesting to note that other common fiber dopant materials (e.g., GeO_2_, P_2_O_5_, and B_2_O_3_) have Raman gain coefficients larger than that of silica. Thus, the YAG-derived fiber results highlighted here suggest that alumina and yttria impart intrinsically lower Raman gain into the glass. An additional benefit, therefore, of these low-silica content yttrium aluminosilicate glasses for optical fibers is a reduced Raman (and Brillouin) gain and therefore increased SRS (and SBS) threshold. 

**Figure 2:**
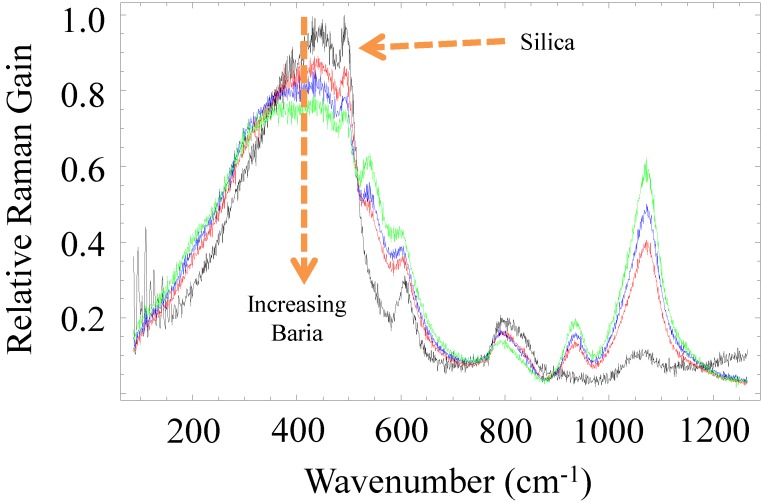
Raman gain spectra (normalized to silica) measured from the barium oxide (BaO) derived fiber for three different baria concentrations. Several new lines appear, likely attributable to the Ba-O bond, including a strong one near 1071 cm^−1^. The silica spectrum (black line) was obtained from the fiber cladding of one of the fibers.

It is important to note that even with the addition of ‘high-Raman-gain’ materials to silica it is still possible to achieve some level of reduction to the Raman gain. As an example, the Raman spectra for the baria-derived fiber [35] are shown in Figure 2. The baria concentrations in the three fibers are 10.4, 12.3, and 18.4 mol%. Relative to the cladding spectrum (pure silica), many of the conclusions about the shape of the spectra obtained from the YAG- and sapphire-derived fiber also seem to hold for the bariosilicate fibers. However, it is also clear that several new lines appear, including a prominent one near 1071 cm^−1^ likely attributable to the Ba-O bond. From the spectra and given the concentrations of baria in the fibers, it can be deduced that the strength of the aforementioned Raman line is relatively large in the glass. In fact, one can deduce by extrapolation that at a baria concentration in the vicinity of 25–30 mol%, the Raman gain will be larger in the baria-derived fiber than that in pure silica. However, at lower BaO concentrations, the contribution to the Raman gain spectrum by baria is at a different phonon frequency, not overlapping with a strong silica phonon line. Thus, as long as the concentration of baria is kept low enough such that its contribution to the Raman spectrum is weaker than silica, the absolute maximum Raman gain coefficient can be reduced. The bottom line is that, in the case of the bario-silicate glasses, the net Raman gain can still be reduced by up to 30% relative to silica.

#### 2.2.3. Optical Fibers with Enhanced Thresholds for Higher Order Mode Instabilities (HOMI)

As noted in the Introduction, there exists in active LMA fibers a power threshold where dynamic randomization of the mode distribution at the laser output is observed, known as “higher order mode instability” (HOMI), and is believed to result from a thermally-induced refractive index grating. Since the process is believed to be driven in part by the thermo-optic coefficient (d*n*/d*T*) the natural question is whether marked increases in the HOMI threshold can be obtained through judicious tailoring of the core material’s thermo-optic coefficient? 

**Figure 3:**
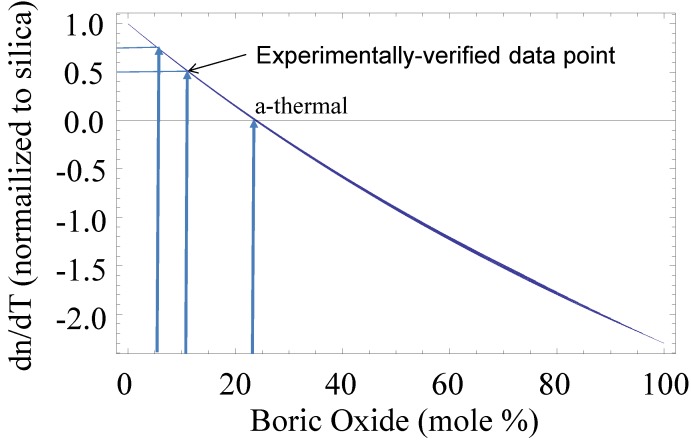
Thermo-optic coefficient of borosilicate glass with increasing boria content. A measured data point on a fabricated fiber is provided. The compositions where d*n*/d*T* reduces by 25% and becomes zero (athermal) are identified on the plot.

While it has already been shown that YAG-derived fibers possess larger thermal conductivities than their silica counterparts [38] (as another potential way to reduce HOMI), the material d*n*/d*T* can also be tailored. Simply stated, if d*n*/d*T* = 0, such modal instabilities should be improved, if not completely removed. Much like the aforementioned work on intrinsically-low-Brillouin-gain glasses, combining materials with thermo-optic coefficients of opposite sign can give rise to a significant reduction in d*n*/d*T*, and possibly even its taking on a value of zero. Materials such as SiO_2_, GeO_2_ (d*n*/d*T* larger than silica), and Al_2_O_3_ (d*n*/d*T* similar to silica) have positive d*n*/d*T*, but this value can also be negative such as in P_2_O_5_ and B_2_O_3_ [39]. As an illustrative example, and based on experimental measurements on B_2_O_3_-doped optical fibers, the thermo-optic coefficient for a borosilicate glass is shown in Figure 3 including a composition where d*n*/d*T* = 0 is identified, along with points where the d*n*/d*T* is 75% and 50% that of silica. The additive materials model was utilized to generate the curve in Figure 3. The data point where d*n*/d*T* reached 50% that of silica is the lowest that the authors have observed to date.

Indeed if the HOMI threshold is proportional to d*n*/d*T*, and if d*n*/d*T* can be zeroed through careful materials selection, then the HOMI problem can, in principle, be negated. However, for the example above, this requires the use of large quantities of boria, which may not be compatible with conventional commercial methods of fiber fabrication. Therefore, targeting more modest increases in the HOMI threshold (25% or 50% for example) enables the use of these conventional fabrication methods, but while still taking advantage of significant improvements in performance. It is worth noting that it is not anticipated nonconventional fiber fabrication methods such as the molten core approach will represent limitations to fiber commercializability in the future. In the particular case of the molten core method, conventional draw processes are employed lessening the obstacles to technology transfer.

#### 2.2.4. Nonlinear Refractive Index and Parasitics Depending Thereon

As noted in the Introduction, processes such as self-phase modulation (SPM) and four-wave mixing (FWM) result from the dependence of the refractive index on the optical intensity (the optical Kerr Effect). As with all other nonlinear optical processes, these may be utilized or be considered parastitics, depending on the requirements of an optical system. FWM and SPM may be useful, for example, for wavelength shifters or for generating of optical solitons, which could be useful for high bit-rate optical communications or short pulse lasers, respectively. However, their broadening and modifying of the optical spectrum may be undesirable in high peak power and multi-wavelength optical amplifier systems. The strength of these interactions is related to the nonlinear refractive index *n*_2_, *i.e*., to the material properties of the glass from which the fibers are made. To first order, the nonlinear index likely carries through the additive model via the refractive index (since, as mentioned above, *n*(*I*)* = n*_0_ *+ n*_2_*I*, where *n*_0_ is the linear refractive index, *I* is the intensity of the optical signal, and *n*_2_ is the nonlinear refractive index) much like the Pockels coefficients do through the strain and stress optic coefficients [22]. To date, however, this has not yet been verified.

There are numerous publications dedicated to the modeling of the nonlinear refractive index [40,41,42]. It is strongly dependent on the linear (nominal) refractive index of the material, and therefore lower refractive index materials tend to have lower *n*_2_ values. An expression for *n*_2_ in terms of widely used glass engineering coefficients, e.g., the Abbe number *ν*_d_ and the refractive index at the Fraunhofer d-line, is [40]

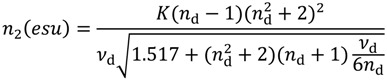
(1)
where *K* is a material constant that is dependent upon the effective oscillator strength (or strength of the induced dipole moment) and shape of the potential well of the oscillator [40]. Upon inspection it is seen that materials with large Abbe numbers and low *n*_d_ are desirable for achieving low nonlinear refractive index values. As a result, classes of fluoride glasses, for example, have relatively low *n*_2_ [42]. Unfortunately, the ranges of Abbe numbers and refractive indices available for most common glass-forming materials are limited (perhaps 50–100 for the former and 1.4 to 1.6 for the latter), and the effectiveness of reducing *n*_2_ in this way is therefore also limited. Of perhaps greater interest is that heretofore a more ‘chemical’ approach may be taken in the design of materials with predetermined *n*_2_. The nonlinear refractive index depends strongly on the polarizability of the material and, therefore, on the nature of the chemical bond (which strongly influence the parameter *K*). It has been found that in some covalent bonding systems, in contrast to ionic ones, the value of *n*_2_ (via *K* [40]) might even take on negative values [43]. Therefore, if an abundance of covalent bonds can be engineered into the material, for example the Al-P bond in the AlPO_4_ system [44], significant reductions in *n*_2_ may be possible. This work has only just begun but offers exciting possibilities to control *n*_2_-based nonlinearities.

### 2.3. Limitations

Knowledge of the appropriate phase behavior does not always guarantee success. As one example of this, consider the system: Yb_2_O_3_-SiO_2_, which could be generalized to the lanthanide oxides. High rare-earth content glasses, particularly silicate glasses for their potential compatibility with silica-based optical fibers, are of interest for their magneto-optic properties. Specifically, high lanthanide oxide silicate glasses could be useful for Faraday isolators in high energy laser systems. 

The Yb_2_O_3_-SiO_2_ phase diagram exhibits a two-liquid immiscibility over a compositional range from about 75–98 mole percent SiO_2_ for temperatures above about 1973K to the upper consolute temperature of 2473 K [45]. In this system a eutectic [L → Yb_2_O_3_ + Yb_2_Si_2_O_7_] exists at a composition of about 38 mole percent SiO_2_ and a temperature of 2125K (1852 °C). The liquidus temperature for this eutectic composition is below the conventional draw temperature for a silica-clad fiber using the molten core approach and so seemed an ideal way to obtain a high Yb-content silicate fiber. Accordingly, homogeneous powders of composition 38 mol% SiO_2_/62 mol% Yb_2_O_3_ were synthesized, pressed into pellets, sleeved into a pure silica tube, and drawn at about 2000 °C. 

**Figure 4:**
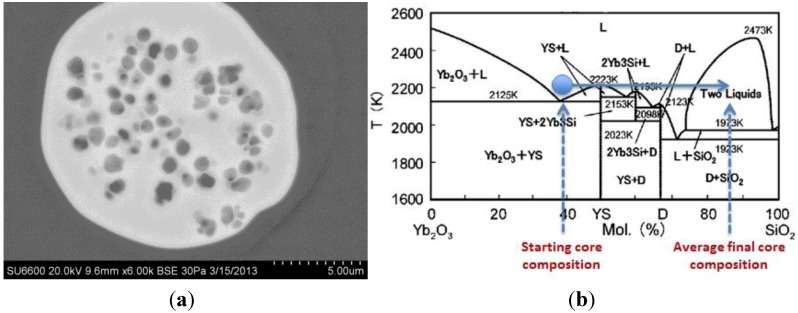
Scanning electron micrograph of the cross-section of a pure-silica clad, Yb_2_O_3_-SiO_2_ core fiber clearly showing (**a**) phase separation and (**b**) the phase diagram for the Yb_2_O_3_-SiO_2_ system (reproduced with permission from [45], Copyright 2005 the Journal of the Ceramic Society of Japan). The phase diagram also depicts the starting core composition and the final average core composition following molten core fiber processing whereby additional SiO_2_ dissolves into the core from the cladding and shifts the final core composition into the two liquid immiscibility region.

Figure 4 provides a scanning electron microscope image of the resultant fiber; clearly observed is phase separation. Upon compositional analysis, the average composition as measured to be about 86 mol% SiO_2_ (~50 weight percent), this SiO_2_ dissolving into the core melt from the pure silica cladding glass. Said composition is right in the center of the aforementioned liquid-liquid immiscibility and the subsequent phase separation was quenched into a solid form as the molten-core fiber cooled. 

### 2.4. A Note on Costs

There is a natural tendency to assume that anything “specialty” is necessarily expensive due, in part, to the lack of an economy of scale associated with something that is not commodity. Here are two specific counter-examples to this assumption.

Described above was an optical fiber derived from sapphire. While sapphire is most commonly thought of in the form of a gem stone, industrial sapphire is quite common and inexpensive. Exemplifying this was the sapphire used in said work [33], which measured 4.2 mm in diameter by 100 mm in length (constituting a weight of 27.5 carats) and cost $175 (USD). The telecommunication-grade silica cladding tube costs about $2,000 (USD), bringing the total materials cost to less than $3,000 (USD; only in optics could silica cost 1100% more than alumina). For comparison, an equivalently-sized (27 carat) sapphire gemstone sold at auction for well over $15,000 (USD); the difference between sapphire as gemstone and sapphire as transparent industrial alumina is clear (pun intended). Obviously one needs a fiber draw tower (but not a lathe) to make the fiber but it was, in the grand scheme of achieving a record 19 dB suppression in SBS, very inexpensive. Photonic crystal fibers, which have not achieved this level of SBS suppression and are far more labor-intensive to make, and cost significantly more in material and labor costs. 

A second example was the recent report of the first glass optical fiber to propagate light via transverse Anderson localization [46,47] as opposed to the conventional total internal reflection. The use of Anderson localization as a propagation modality opens the door to the use of optical fibers for spatially multiplexed high data capacity information links. The glass used in that work was drawn from a porous artisan glass known as “satin quartz”, which costs $75 (USD) for a meter-length rod. As with the sapphire, one still needs a draw tower (but no lathe) and the appropriate know-how and expertise but those are constant no matter what is being drawn. 

From the perspective of material and labor costs, these specialty fibers can be remarkably inexpensive. The difference is not cost but understanding of the enabling materials and glass science and chutzpah to try something new. 

## 3. Experimental Section

The specific optical fibers whose properties are described herein have been reported on individually. For detailed information on the material and fiber fabrication and characterization, the reader is referred to the following References: YAG-derived fibers [24,34], sapphire-derived fiber [33], spinel-derived fiber [36], baria-derived fiber [35], ytterbia-derived fiber [48], and lanthana-derived fiber [49]. 

More generally, though, optical fibers conventionally are prepared via one of a series of chemical vapor deposition methods [50,51,52]. In such cases, the core and clad glasses possess reasonably similar thermal (particularly glass transition temperatures, *T*_g_) and thermomechanical properties such that they can be co-drawn into a waveguiding fiber that possesses high strength and appropriate optical properties. This thermal and thermomechanical compatibility tends to imply that the compositions of said core and clad glasses are not overly different. 

However, as noted above, most of the fibers treated in this work possess compositions that suffer from phase instabilities, such as liquid-liquid immiscibility, and so cannot be processed using the aforementioned conventional measures. As such, a new method had to be developed that yielded high quality optical fibers while still being industrially relevant. Based on the original work of Reference [53], the molten core approach was employed. References [4,54] provides a more detailed review of the molten core method and material solutions primarily for SBS and SRS mitigation but does not address issues associated with SPM, FWM, and HOMI (which is discussed in this work). Briefly, instead of relying on core/clad material compatibility, the molten core approach requires that the core phase melt at a temperature below where the cladding phase draws into fiber; in other words, during the fiber fabrication, the core is above its liquidus (or upper consulate point if an immiscibility exists). Silica is a preferred cladding glass for the molten core approach not only because it facilitates compatibility of the molten core-derived fibers with conventional silica-based fibers, but also it is a highly refractory glass such that many core phases exist that melt at temperatures below the draw temperature for pure silica (ranging from about 1925 to 2050 °C, depending on size of silica preform).

While advantageous in many cases, the cladding glass is attacked by the core melt and is somewhat dissolved. This makes the final post-drawn fiber an interaction product of the initial core phase and the cladding glass, depending upon the draw time and temperature as well as preform and fiber dimensions. In the specific case of SBS suppression, where most of the core phases chosen possess negative photoelastic constants, the dissolution of the silica (possessing a positive photoelastic constant) can give rise to a resultant multi-component glass with near-zero photoelasticity. 

## 4. Conclusions

Provided herein has been a review of the present state of next generation optical fibers that meet the needs of present and future high energy laser systems from the specific perspective of SBS and SRS mitigation through a purely materials approach. Simple materials systems, including those in the Al_2_O_3_-SiO_2_, BaO-SiO_2_, Al_2_O_3_-Y_2_O_3_-SiO_2_, and Al_2_O_3_-MgO-SiO_2_ families, have been shown to yield optical fibers of classic core/clad geometries that exhibit reductions in SBS by nearly 20 dB and SRS by about 3 dB, though much work remains. Also described were fibers with marked increases in the threshold for higher order mode instabilities (HOMI) though judicious tailoring of the core materials’ thermo-optic coefficient, d*n/*d*T*. 

Though most of the focus here has been placed on crystal-derived all-glass optical fibers fabricated using the cost-effective and industrially compatible molten core approach, the more general message is that materials clearly offer solutions to present and future problems plaguing the high energy fiber-based laser sector. Accordingly, significant opportunities exist for the optical materials community in terms of scholarly topics for study, funding to do so, and job prospects. 
